# Comprehensive Analysis of Subcellular Localization, Immune Function and Role in Bacterial wilt Disease Resistance of *Solanum lycopersicum* Linn. ROP Family Small GTPases

**DOI:** 10.3390/ijms23179727

**Published:** 2022-08-27

**Authors:** Qiong Wang, Dan Zhang, Chaochao Liu, Yuying Li, Yanni Miao

**Affiliations:** 1 School of Horticulture and Plant Protection, Yangzhou University, Yangzhou 225009, China; 2 School of Biotechnology, Jiangsu University of Science and Technology, Zhenjiang 212021, China; 3 Lingnan Guangdong Laboratory of Modern Agriculture, Genome Analysis Laboratory of the Ministry of Agriculture, Agricultural Genomics Institute at Shenzhen, Chinese Academy of Agricultural Sciences, Shenzhen 440307, China

**Keywords:** tomato, Rho-related proteins, polybasic region, immune response, *Ralstonia solanacearum*

## Abstract

ROPs (Rho-like GTPases from plants) belong to the Rho-GTPase subfamily and serve as molecular switches for regulating diverse cellular events, including morphogenesis and stress responses. However, the immune functions of ROPs in *Solanum lycopersicum* Linn. (tomato) is still largely unclear. The tomato genome contains nine genes encoding ROP-type small GTPase family proteins (namely *SlRop1–9*) that fall into five distinct groups as revealed by phylogenetic tree. We studied the subcellular localization and immune response induction of nine SlRops by using a transient overexpression system in *Nicotiana benthamiana* Domin. Except for SlRop1 and SlRop3, which are solely localized at the plasma membrane, most of the remaining ROPs have additional nuclear and/or cytoplasmic distributions. We also revealed that the number of basic residues in the polybasic region of ROPs tends to be correlated with their membrane accumulation. Though nine SlRops are highly conserved at the RHO (Ras Homology) domains, only seven constitutively active forms of SlRops were able to trigger hypersensitive responses. Furthermore, we analyzed the tissue-specific expression patterns of nine *ROPs* and found that the expression levels of *SlRop3*, *4* and *6* were generally high in different tissues. The expression levels of *SlRop1*, *2* and *7* significantly decreased in tomato seedlings after infection with *Ralstonia solanacearum* (E.F. Smith) Yabuuchi et al. (GMI1000); the others did not respond. Infection assays among nine ROPs showed that SlRop3 and SlRop4 might be positive regulators of tomato bacterial wilt disease resistance, whereas the rest of the ROPs may not contribute to defense. Our study provides systematic evidence of tomato Rho-related small GTPases for localization, immune response, and disease resistance.

## 1. Introduction

*Ralstonia solanacearum* (E.F. Smith) Yabuuchi et al. (*R. solanacearum*) is one of the most devastating plant pathogenic bacteria, causing bacterial wilt disease worldwide [1]. *R. solanacearum* populates soils, infects plants through their roots, thrives in the xylem vessels, which transport water to various organs, eventually leading to typical wilting symptoms and even the rapid death of the hosts [2]. As a soil-borne pathogen, *R. solanacearum* can invade a broad range of plant species, including many economical crops, such as *Nicotiana benthamiana* Domin. (tobacco), *Arachis hypogaea* Linn. (peanut), *Musa nana* Lour. (banana), *Capsicum annuum* Linn. (pepper), *Solanum tuberosum* Linn. (potato), and *Solanum lycopersicum* Linn. (tomato) [3]. Tomato is an important and favourite vegetable crop worldwide. The ripening fruits of tomato are rich in vitamin C, lycopene, organic acids, and other healthy nutrients [4]. Since tomato production is seriously threatened by bacterial wilt disease, breeding of disease-resistant tomato varieties is a vital research objective. However, bacterial wilt disease resistance-related genes are still poorly identified.

Small GTPases are a highly conserved class of proteins in eukaryotes with multifunctions that regulate cellular signal transduction, membrane trafficking, nucleocytoplasmic transport, growth, and development [5]. These proteins serve as molecular switches for the transitions between inactive (GDP-bound) and active (GTP-bound) forms. Normally, GDP is tightly bound, and GTP is hydrolysed very slowly; thus, small GTPases require help from other components: (1) guanine nucleotide exchange factors (GEFs) that facilitate GDP dissociation; (2) GTPase activating proteins (GAPs) that stimulate the intrinsic GTP hydrolysis; (3) guanine dissociation inhibitors (GDIs) that form a soluble complex with small GTPases as their GTP/GDP alternations are related to membrane/cytosol alternations [6,7]. In plants, there are four main clades of small GTPases, including Ran GTPases, Rab GTPases, Arf GTPases, and Rho-like GTPases of plants (ROPs) [8]. Notably, ROPs are plant-specific and composed of unique Rac/Rop-related small G proteins, which have been widely found to be involved in root-hair development, phytohormone responses, pollen tube growth, and innate immunity [9,10].

Accumulating evidence suggests that Rho-like small G proteins are indispensable regulators of immune signaling in plants [11]. OsRac1 is a positive regulator in rice immunity, acting downstream of both pattern recognition receptors (OsCERK1) and resistance proteins (Pit, PID3, and Pia) [12,13,14,15,16,17]. OsRac1 forms complexes with many interactors, including OsMPK3/6, the ROS scavenger OsMT2b, NADPH oxidase, the scaffolding protein OsRACK1A, the heat shock proteins Hsp90/70, and the lignin biosynthesis enzyme OsCCR1, to regulate various aspects of immune responses [18,19,20,21,22,23,24]. In addition, the DOCK family GEF OsSPK1 has been shown to activate OsRac1 directly, and the OsSPK1-OsRac1-dependent signaling module generally plays a critical role in rice immunity [25,26]. Overexpressing the inactive form of the OsRac1 mutant in tobacco disrupted the ROS production induced by Tomato Mosaic Virus (TMV), indicating that the tobacco OsRac1 cognate protein may also be involved in defense against pathogens [27]. The dominant positive Racs from Zea mays induce ROS burst in mammalian cells [28]. Recently, researchers found that SlROP-II.1 (hereafter named SlRop3), CaROP-II.1 (hereafter named CaRop8), and NbROP-II.1 show resistance against *Phytophthora capsici* Leonian (*P. capsici*) in tomato, pepper, and tobacco, respectively [29]. Nevertheless, OsRac4 and OsRac5 negatively regulate disease resistance to blast fungus [15]. In barley, three members (HvRACB, HvRAC3, and HvROP6) of Rac/Rop family negatively regulate disease resistance to powdery mildew fungi by facilitating pathogen accessibility [30,31]. Stable and transient expression of the dominant-negative mutant (DN-AtRop1) from *Arabidopsis thaliana* (Linn.) Heynh. (Arabidopsis) promotes ROS production and inhibits the growth of *Phytophthora infestans* (Mont.) de Bary (*P. infestans*) in potato [32]. Similarly, overexpression of wild-type and DN-CaRop1 in tobacco conferred resistance to *R. solanacearum* and aphids by activating cell death during infection [33]. Moreover, TaRop10, the group II ROP from wheat, showed negative regulation of wheat immunity against stripe rust [34]. In addition, tobacco NtRHO1 plays a negative role in defense response to TMV [35]. In conclusion, Rho-related small GTPases perform a dual function via a variety of signal transductions during pathogen infections. However, the function of tomato Rop/Rac family small G proteins in resistance to bacterial wilt disease has not yet been demonstrated.

Membrane association is essential for plant-specific ROP functions and achieved by two posttranslational lipid modifications [36]. A considerable number of Rho-related GTPases contain a hypervariable C-terminal tail consisting of abundant Cys motifs and basic residues (lysine or arginine, K/R), thus termed the polybasic region (PBR) [37]. According to the amino acid sequences of PBR, ROPs are classified into two major subgroups: (1) type I, terminating with a conserved CaaL (a, aliphatic amino acid) box motif in which cysteine is the prenylation site; (2) type II, lacking the CaaL motif but containing a GC-CG box in which cysteine undergoes S-acylation [38]. Deletion or mutation of the GC-CG sequence of AtROP10 disrupted its plasma membrane localization, and the truncation of the polybasic domain caused the same consequences [37]. To date, the systematic analysis of the relationship between PBRs and membrane attachment have been characterized in Arabidopsis, wheat, and rice [15,30,37], but there are no reports of this relationship for tomato ROPs. 

In this paper, we first identified the ROP family small GTPases and analyzed the evolutionary relationships of nine SlRops with ROPs in other plant species. Subcellular localization, tissue-specific expression patterns, and involvement in defense against tomato bacterial wilt disease of SlRop1–9 were determined. We revealed that the membrane targeting of tomato ROPs tend to be correlated with the contents of basic residues in the PBR region. Most constitutively active forms of SlRops, except SlRop1 and 2, activate significant hypersensitive responses when they are transiently overexpressed in *Nicotiana benthamiana* Domin. (*N. benthamiana*). Nonetheless, only SlRop3 and 4 make contributions to block the colonization of *R. solanacearum* in tobacco leaves, whereas the remaining SlRops may not be involved in defense. Collectively, our findings provide basic reference information for deciphering the molecular mechanisms how tomato interacts with *R. solanacearum* and the breeding of novel varieties that are highly resistant to tomato bacterial wilt disease.

## 2. Results

### 2.1. Identification and Phylogenetic Analysis of SlRops

**Figure 1:**
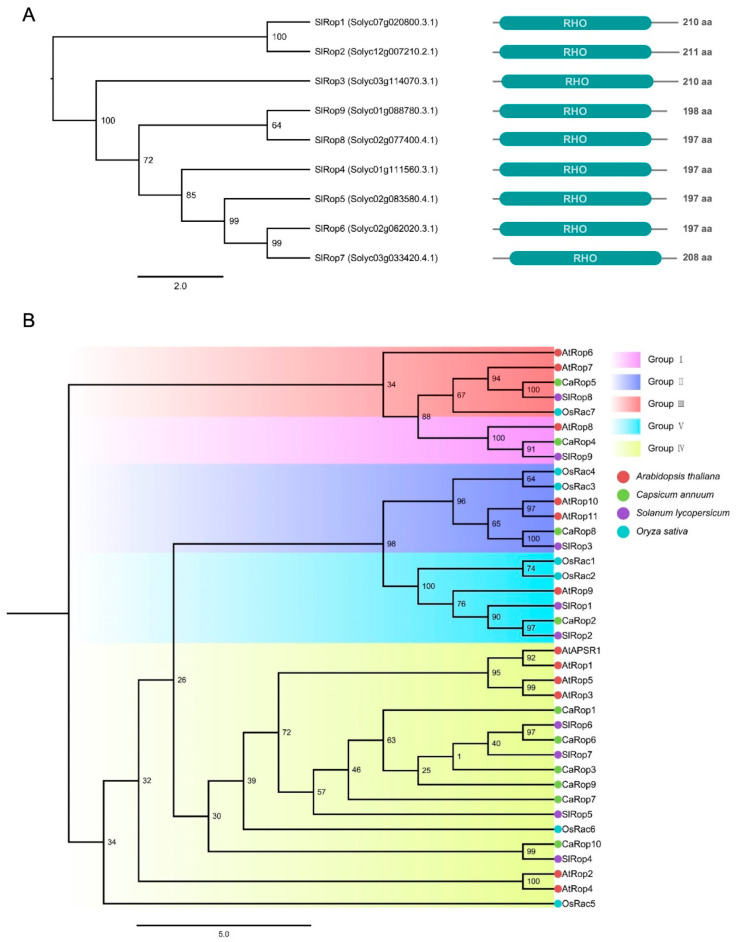
Phylogeny of SlRops identified in *Solanum lycopersicum* (**A**), Phylogenetic and characteristic analysis of nine Rops from the tomato genome database. Left panel, the phylogenetic tree constructed using the protein sequences of nine genes with IQ-TREE. A multiple protein sequence alignment was generated using the MUSCLE tool (Edgar 2004), followed by trimming using TrimAI. The numbers at each node indicate the bootstrap support value calculated from one thousand replicate settings. Right panel, the corresponding schematic diagram of the protein domain structure predicted by SMART. The protein length of each ROP is shown on the right. aa, abbreviation for amino acid. Cyan boxes indicate the length and location of the canonical RHO domain within the Rac/Rop family small GTPases. (**B**), The evolutionary relationship among Rho-like proteins from *Solanum lycopersicum*, *Arabidopsis thaliana*, *Capsicum annuum*, and *Oryza sativa*. The phylogenetic tree was obtained similarly to that described in A. Five groups are separately presented with magenta (Group I), blue (Group II), red (Group III), yellow (Group IV), and cyan (Group V).

To identify the Rop/Rac subfamily small GTPases in *Solanum lycopersicum* L. (tomato), we used the rice OsRac1 protein sequence as a query for a BLAST search against the tomato genome database at the Sol Genomics Network (https://solgenomics.net/, accessed on 13 August 2021). The genome-wide screening results showed that there are nine proteins displaying over 75% shared identity with OsRac1 and possessing a canonical RHO domain that is highly conserved in Rop/Rac small GTPases among different plant species (Figure 1A). A multiple amino acid sequence alignment revealed that these nine proteins have very high similarity to each other, and all contain conserved functional domains for GTP hydrolysis, GDP/GTP binding, etc. (Figure S1). Therefore, we termed these nine proteins SlRop1–9 (Figure 1A), and their gene IDs are consistent with those reported previously [29]. *SlRop1*–*9* were located on 5 different chromosomes: *SlRop4* and *9* on chromosome 1; *SlRop5*, *6*, and *8* on chromosome 2; *SlRop3* and *7* on chromosome 3; *SlRop1* on chromosome 7; and *SlRop2* on chromosome 12 (Figure S2A). Next, we evaluated the evolutionary relationship of nine SlRops in tomato, seven OsRacs in rice, eleven AtRops and one Rop-activity possessing protein AtAPSR1 in Arabidopsis, and ten CaRops in pepper (Figure 1B). The phylogenetic tree was generated by using IQ-TREE with one thousand bootstrap replicates. All the Rac/Rop small GTPases shown in this phylogenetic tree were divided into five groups: Group I, including three Rops; Group II, including eight Rac/Rops; Group III, including four Rac/Rops; Group IV, including eighteen Rac/Rops; and Group V, including four Rops. Remarkably, only SlRop3 (also known as SlROP-II.1) falls into the same group as OsRac1, a key regulator of rice immunity [14,15,16,39]. Additionally, SlRop4, 5 and 6 fall into the same group as CaRop1, AtRop1, AtRop6, OsRac5, and OsRac6, which also respond to pathogen or aphid attacks [15,32,33,40]. Therefore, it is possible that certain SlRops play important roles in tomato innate immunity.

### 2.2. Relationship between the Polybasic Region and Subcellular Localization of SlRops

**Figure 2:**
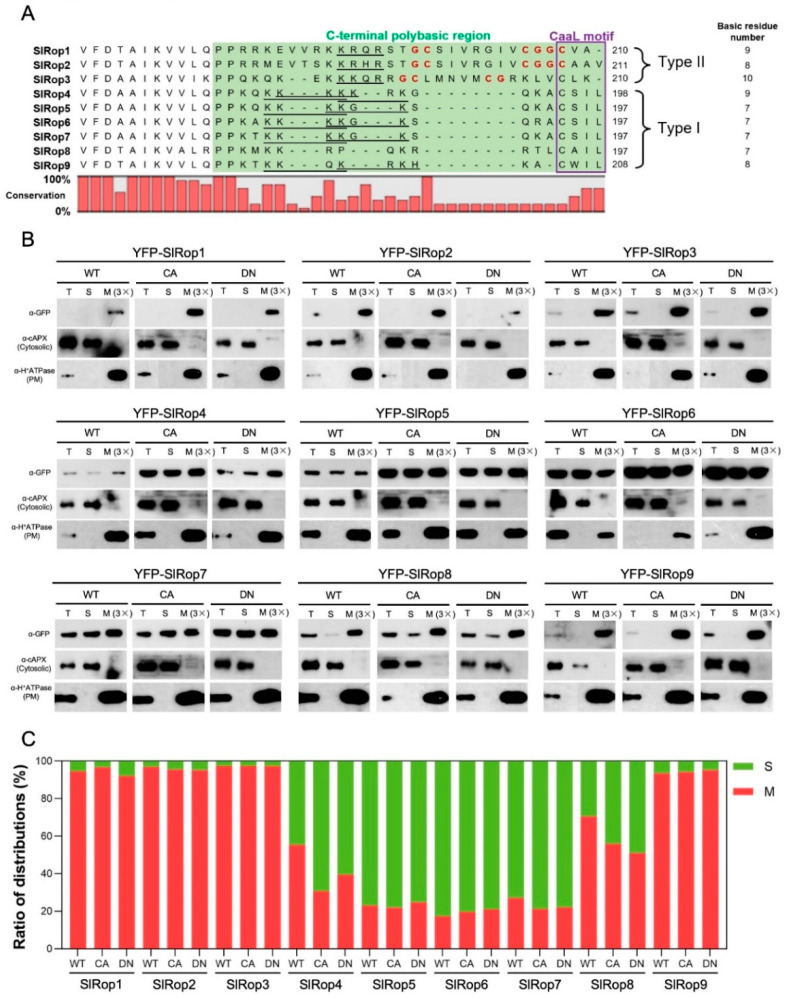
Analysis of the PBRs and subcellular localization of SlRop1–9. (**A**), Amino acid sequence alignment of the C-terminal polybasic regions (PBRs) of tomato Rac/Rop family proteins. The C-terminal PBRs are highlighted with a green background. The purple box indicates the position of the CaaL motif. Red bold letters represent GC-CG box motif. The NLS sequences are underlined. The numbers on the right denote the total basic residues in PBRs. The nine Rops in tomato are subdivided into two types (Type I: SlRop4–9; Type II: SlRop1–3) based on whether they contain the CaaL motif. (**B**), Subcellular distribution of tomato Rho-like small GTPases in *N. benthamiana*. Agrobacterium carrying YFP-fused SlRop1–9 wild type and mutants were individually injected into tobacco leaves. WT, wild type; CA, constitutively active; DN, dominant-negative; PM: plasma membrane; T: total extract, S: soluble fraction, M: microsomal fraction. M (3×) indicates three times enrichment relative to S. Immunoblotting was performed with anti-GFP (for SlRops), anti-H^+^ATPase (PM marker), and anti-cAPX (cytoplasm marker) antibodies. (**C**), Ratio of membrane and cytosolic distributions of YFP-SlRops. Red and green bars indicate the percentage of membrane and cytosolic fractions, respectively. S: soluble fraction, M: microsomal fraction. The experiment was repeated twice.

Previously published papers showed that many Ras and Rho-type small GTPases have a C-terminal polybasic region (PBR), which is critical for membrane attachment and protein-protein interactions [15,41]. A protein sequence analysis of the SlRop1–9 C-terminus revealed that all Rho subfamily small GTPases in tomato possess PBRs (Figure 2A). Upon further analysis of the PBRs in nine SlRops, we found that SlRop1–9 can be subdivided into two types in terms of a canonical CaaL motif relevant to prenylation: SlRop4–9 belong to type I because they contain a conserved CaaL motif at the C-terminus, although SlRop1–3 belong to type II since they lack an intact CaaL motif (Figure 2A). In contrast, instead of type-I SlRop4–9, type-II SlRop1–3 all contain a conserved GC-CG box separated by several nonpolar amino acids, as reported previously, which are quite important for the association of type-II Rho family small G proteins with the plasma membrane [37,38]. An analysis of the amino acid composition of PBRs showed that SlRop1, 3 and 4 are rich in basic residues (K/R/H), indicating that these three proteins may prominently accumulate at the cell membrane. Moreover, the PBRs of most SlRops except SlRop8 all harbor a K-b-x-b (b, basic amino acid; x, random amino acid) sequence for nuclear localization (NLS). Taken together, our analysis of the PBRs from nine tomato Rho-type small GTPases suggests that they probably localize at the plasma membrane and nucleus.

To verify our speculation, we first investigated the subcellular localization of nine tomato Rops by using a transient expression system in *N. benthamiana*. The coding sequences of nine SlRops were individually fused with yellow fluorescent protein (YFP) at the N-terminus, driven by the 35S promoter, transiently overexpressed in tobacco leaves, and detected under a confocal microscope. As a result, all the YFP-tagged Rops in tomato were predominantly observed around the plasma membrane (Figure S4). Except for SlRop1 and 3, the remaining SlRops, including whole Rops from type I and SlRop2 from type II, were also found to be clearly localized in the nucleus (Figure S4). It was mystifying that the in vivo localizations of SlRops were not completely consistent with our predictions based on sequence analysis data. Interestingly though, SlRop2 terminates with CAAV, closely resembling the CaaL motif unique to the type-II Rac/Rops (Figure 2A). This finding raises the possibility that the integrity of the C-terminal Caax sequence might be important for importing Rho subfamily proteins to the nucleus. To prove this speculation, we generated the SlRop3 + L mutant which contains the intact CaaL motif, and then checked its subcellular localization. Unfortunately, this mutant displayed a similar distribution to the WT and did not show nuclear signals (Figure S5A) since the cytoplasm was packed tightly against the plasma membrane in tobacco cells and it was technically difficult to determine conclusively the localization of SlRops. Therefore, we undertook biochemical fractionation to clarify SlRops’ localization. We overexpressed YFP-tagged SlRop1–9 WT, constitutively-active (CA) and dominant-negative (DN) mutants in *N. benthamiana* and roughly fractionated the total proteins of tobacco leaves into two compartments: soluble (S) and membrane (M) fractions. The results showed that SlRop1, 2, 3 and 9 were only detected in the membrane fraction, although the remaining SlRops had both membrane and cytosolic distributions (Figure 2B,C). The localization patterns of CA-/DN-SlRops showed basically similar trends as those of the wild-type SlRops, suggesting that the activation states of Rho-related small GTPases in tomato did not appear to affect their subcellular localization (Figure 2B,C). Taken together, SlRop1 and SlRop3 are solely localized at the plasma membrane; SlRop2 and SlRop9 are localized in both plasma membrane and nuclear; the remaining SlRops have plasma membrane, cytoplasmic and nuclear distributions.

**Figure 3:**
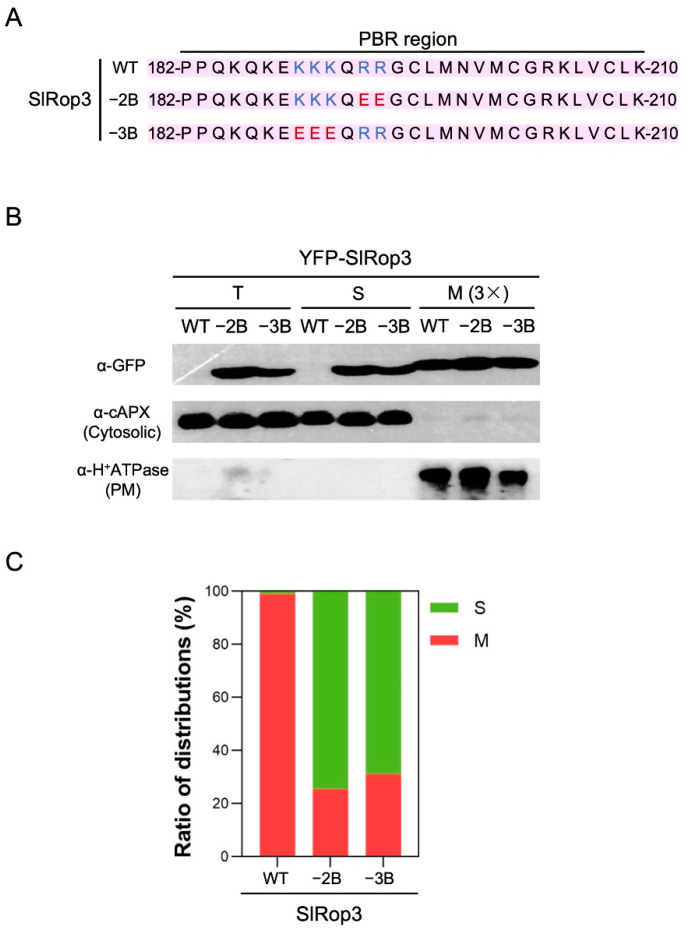
The correlation between the number of basic residues in the PBRs and subcellular localization. (**A**), illustration of the positions of basic amino acid substitutions in the PBR region of SlRop3. −2B and −3B indicate two and three basic residues were mutated to acidic glutamic acid (E), respectively. Blue and red letters represent selected basic residues without and with mutations, respectively. (**B**), Subcellular accumulation of SlRop3 mutants in *N. benthamiana*. YFP-fused SlRop3 wild type and mutants were overexpressed in tobacco leaves. PM: plasma membrane; T: total extract, S: soluble fraction, M: microsomal fraction. M (3×) indicates three times enrichment relative to S. Immunoblotting was performed with anti-GFP (for SlRop3), anti-H^+^ATPase (PM marker), and anti-cAPX (cytoplasm marker) antibodies. (**C**), Ratio of membrane and cytosolic distributions of YFP-SlRop3 mutants. Red and green bars indicate the percentage of membrane and cytosolic fractions, respectively. S: soluble fraction, M: microsomal fraction. The experiment was repeated twice.

Coincidentally, the membrane distribution of SlRop5–7 with minimum basic residues in the PBRs among nine ROPs only accounted for a small proportion; although SlRop1 and SlRop3, which contain the top two most enriched basic amino acids within PBRs, were predominantly localized at the plasma membrane (Figure 2C). These data support the previous paper which pointed out that there is a strong correlation between the subcellular localization and the number of basic residues in the PBR region of plant Rho GTPases [15]. To solidly prove this conclusion, we selected SlRop3 which contains ten basic residues in its PBR region and decreased the number of basic residues in SlRop3 by site-directed mutagenesis (Figure 3A). Then we fractionated the cellular components of *N. benthamiana* leaves overexpressing YFP-tagged SlRop3 WT or mutants. As a result, the percentage of membrane accumulation in SlRop3 mutants was clearly decreased compared to that in WT (Figure 3B,C). This result confirmed the previous conclusion that the membrane association and the basic residues content in the PBRs were likely to be positively correlated.

### 2.3. Involvement of SlRops in Hypersensitive Response (HR) Induction

**Figure 4:**
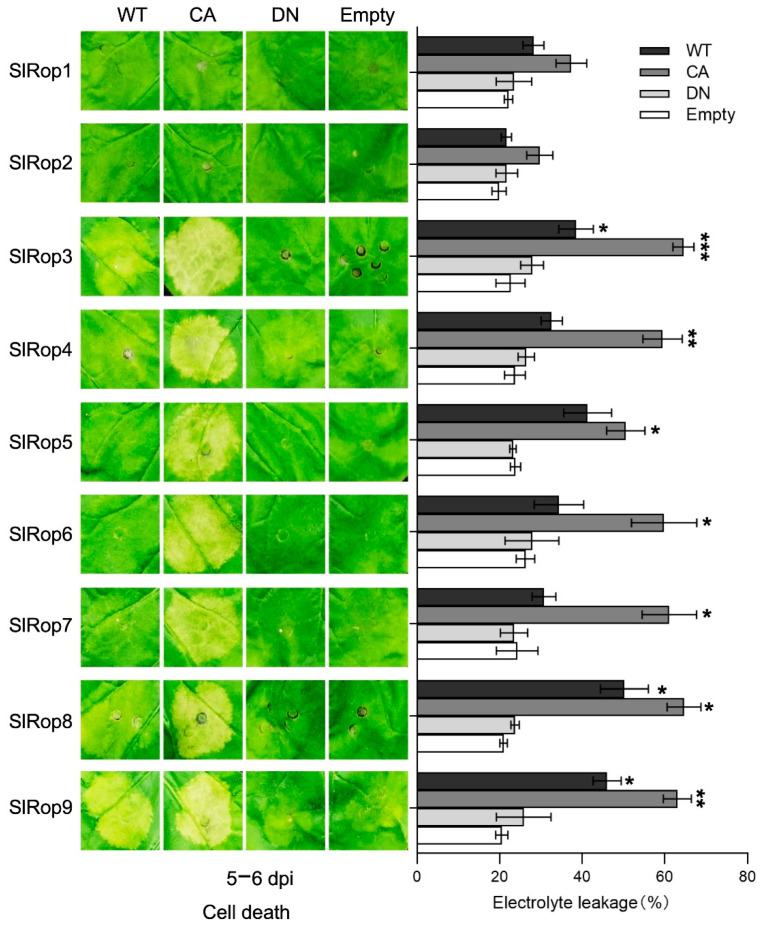
Members of SlRops are involved in cell death induction. Analysis on the inducibility of tomato ROPs for cell death in *N. benthamiana*. Nine free-tagged SlRops with different activation levels were transiently expressed in tobacco. The left-side pictures showing the injected regions on the leaves were captured at 5 dpi. WT, wild type; CA, constitutively active; DN, dominant-negative. The statistical histogram on the right displays the quantification of cell death by measuring the percentage of electrolyte leakage. Data are the average ± standard deviation (SD) of three independent experiments by one-way analysis of variance (ANOVA) (* *p* < 0.05; ** *p* < 0.01; *** *p* < 0.001). The experiments were independently performed at least three times.

To examine the HR-inducing abilities of Rops in tomato, WT, CA, and DN forms of SlRop1–9 were properly agroinfiltrated into *N. benthamiana* leaves. By five days post-inoculation (dpi), active SlRop3–9 mutants clearly produced cell death in the absence of pathogens; wild-type SlRop3, 4, 8, and 9 also triggered weaker cell death compared with that in their CA form; and dominant-negative mutants of all SlRops had no obvious symptoms (Figure 4). Notably, both SlRop1 and 2 failed to induce cell death regardless of their activation state, even though these plants were cultured over ten days after injection. To assess the level of cell death induced by nine SlRops precisely, we employed an electrolyte leakage assay to quantify it. The statistical analysis of ion leakage from dead cells was consistent with phenotypes that occurred on the tobacco leaves.

**Figure 5:**
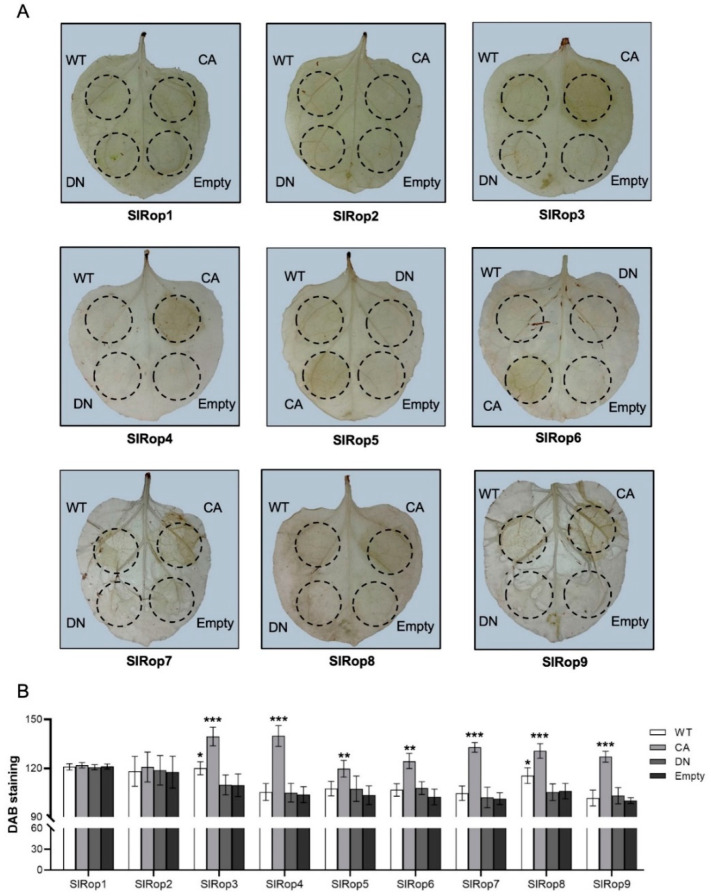
Role of tomato Rac/Rop-related small GTPases in ROS production. Nine free-tagged SlRops with different activation levels were transiently expressed in *N. benthamiana* to examine their ability to produce ROS. By 5 dpi, the agroinfiltrated leaves were stained with DAB, decolored using absolute ethanol. WT, wild type; CA, constitutively active; DN, dominant-negative; Empty, empty vector used as a negative control. (**A**), Photograph showed ROS production in situ. (**B**), Quantitative analysis of ROS production. Bars indicate DAB staining intensity relative to that observed after infiltration with negative control. Data are expressed as mean ± SD (one-way ANOVA; * *p* < 0.05; ** *p* < 0.01; *** *p* < 0.001; *n* = 8).

ROS burst is a hallmark response of plant immunity and is induced primarily by RBOH family proteins [42]. Therefore, we also checked ROS production by staining the infiltrated tobacco leaves using 3,3′-diaminobenzidine (DAB). As expected, apart from SlRop1 and 2, the active form of the rest markedly produced ROS at 5 dpi; WT-SlRop3 and SlRop8 induced a low ROS burst (Figure 5). ROS production was accompanied by cell death in most cases. To exclude the possibility that protein expression levels in tobacco affected HR induction, all of the SlRops tested above were expressed and detected by western blotting using an anti-GFP antibody. The results showed that all SlRop1–9 WT and mutants were expressed well in *N. benthamiana* (Figure S5B). In conclusion, activated SllRop3–9 are all able to promote cell death and ROS production, but SlRop1 and 2 are not. In other words, SlRop3–9 are likely to be involved in immune signaling.

### 2.4. Tissue-Specific Expression Patterns of SlRops

**Figure 6:**
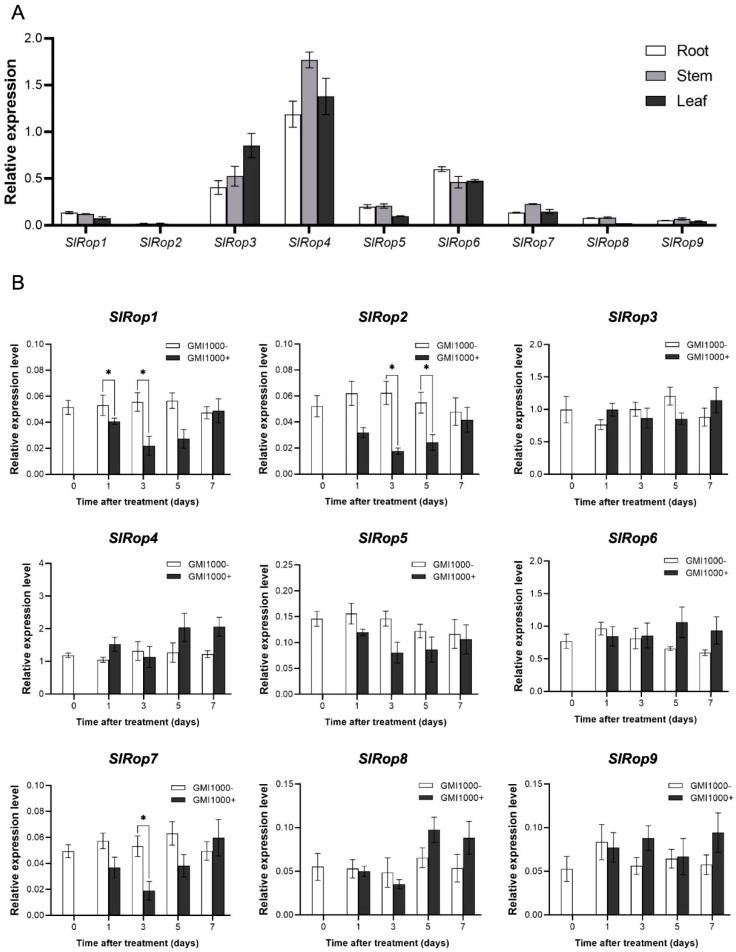
Transcript levels of *SlRop1*–*9* in different tissues and upon *R. solanacearum* infection. (**A**), Natural tissue-specific expression of Rho-like small GTPases in tomato. Total mRNAs were extracted from the roots, stems, and leaves of tomato seedlings. qRT-PCR was performed with specific primers for *SlRop1*–*9* and *ubiquitin*. The relative expression levels of *SlRops* were calculated using *ubiquitin* as the internal reference gene. Data are represented as the means ± SD. Three independent experiments were conducted. (**B**), The relative transcriptions of nine genes from tomato after infection with *R. solanacearum*. The cDNAs from whole tomato seedlings treated with the *R. solanacearum* GMI1000 strain at different time points (1, 3, 5, and 7 d) were used as templates for *SlRop1*–*9* amplification. Water treatment was used as the negative control. *Ubiquitin* was selected as the internal reference for analyzing the relative expression levels of target genes. Data are expressed as the means ± SD (one-way ANOVA; * *p* < 0.05; *n* = 3).

Next, we tested the expression profiles of *SlRops* in tomato plants by using quantitative real-time PCR with corresponding specific primer sets for nine *SlRops*. Because our research focused on tomato bacterial wilt disease, we only isolated the total RNA from the roots, stems, and leaves of tomato seedlings. As we all know, the root-stem-leaf is regarded as the invasion route of *Ralstonia solanacearum* from soil to tomato. Overall, there were no significant specific expression preferences for the nine *SlRops* among the three tissues. *SlRop3, 4,* and *6* had ubiquitously higher expression levels than the other *SlRops*. In addition, *SlRop2* was barely detectable in all tissues (Figure 6A).

To determine the potential role of tomato Rho-type small GTPases in resistance to bacterial wilt disease, we measured the mRNA accumulation levels of *SlRop1*–*9* after *R. solanacearum* GMI1000 strain inoculation at different time points. As shown in Figure 6B, the expression levels of *SlRop1*, *2*, and *7* significantly decreased compared to the negative control at 1 or 3 dpi, and the expression levels of the remaining *SlRops* did not alter upon treatment. These results raised two possibilities: (1) SlRop1, 2, and 7 play negative roles in resistance against *R. solanacearum*, but others do not contribute to defense; (2) activation states rather than expression levels of SlRops may be changed during infection, which is more essential for disease resistance. Meanwhile, we also evaluated the pathogenicity of GMI1000 on tomato cultivar *Ailsa Craig*. The infected seedlings started to show wilting symptoms from the 2 dpi and after seven days, all of them were wilted or dead (Figure S6). This result suggested that tomato cultivar *Ailsa Craig* is susceptible to *R. solanacearum* GMI1000.

### 2.5. Roles of SlRops in Disease Resistance to Tomato Bacterial Wilt

**Figure 7:**
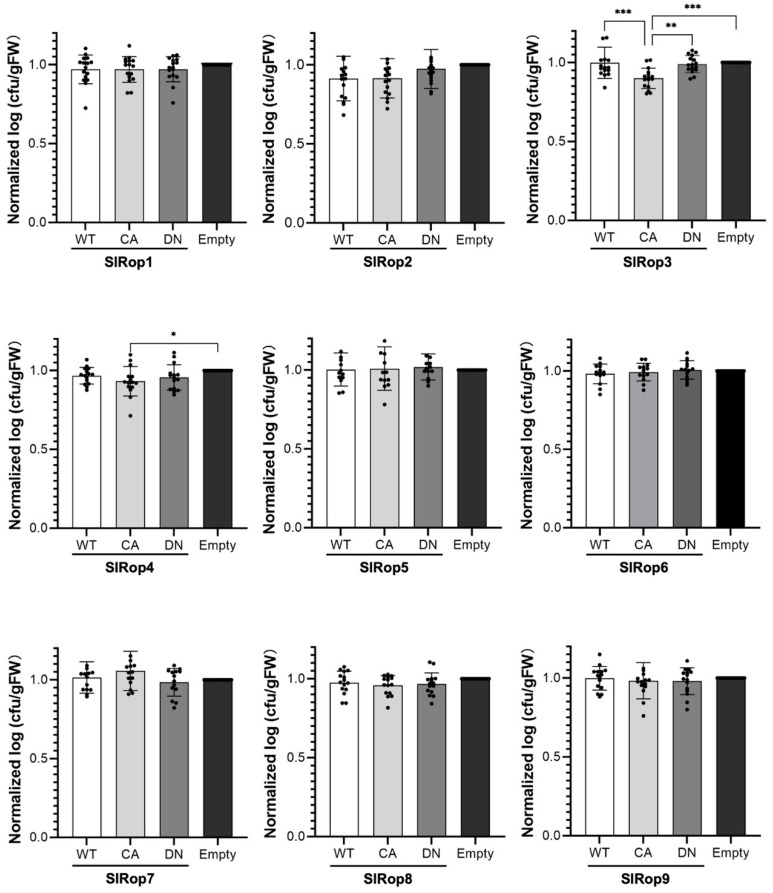
Contribution of SlRop1–9 to defense against *R. solanacearum.* Effects of tomato ROPs on the virulence of *R. solanacearum* in *N. benthamiana*. The *R. solanacearum* Y45 strain was injected into the regions overexpressing different activation states of 35S promoter-driven SlRop1–9. By 2 dpi, the extractions from inoculated leaves were spread on the medium. The number of bacteria towards plant fresh weight was calculated and then converted to a logarithmic scale. Bars indicate the growth of Y45 relative to that determined in negative control Empty. WT, wild type; CA, constitutively active; DN, dominant-negative; Empty, empty vector. Data are expressed as means ± SD (one-way ANOVA; * *p* < 0.05; ** *p* < 0.01; *** *p* < 0.001). The relative replication of bacteria (Empty = 1) is shown. Black dots in the charts indicate each normalized value including biological and technical replicate. The independent experiments were repeated at least three times.

Recently, Dr. Macho’s group developed a fast and easy method for studying the effects of transient gene overexpression or silencing on *Ralstonia solanacearum* virulence in *N. benthamiana* leaves [43]. In this system, the *R. solanacearum* Y45 strain lacking two effector proteins that are recognized by *N. benthamiana* immunity and carrying tetracycline resistance was used. We transiently expressed the WT/CA/DN forms of SlRop1–9 and an empty vector independently. Then, Y45 was infiltrated into the same region that was injected with Agrobacterium on the next day. By 2 dpi, we started to measure the biomass of *R. solanacearum* in different samples. Ultimately, we found that overexpressing active SlRop3 and SlRop4 significantly suppressed the replication of *R. solanacearum* and the remaining Rops had no impact on the growth and proliferation of this pathogen (Figure 7). Furthermore, SlRop3 makes more contribution to tomato bacterial wilt disease resistance than the others (Figure 7). In summary, SlRop3 is a positive regulator of tomato bacterial wilt disease resistance. SlRop4 may be a minor factor in defense against *R. solanacearum*, whereas other members of the tomato ROP subfamily small G proteins are not involved in disease resistance to bacterial wilt under our experimental conditions.

## 3. Discussion

To date, seven Racs in rice, eleven Rops in Arabidopsis, six Rac/Rops in barley, and sixty-six Rops in *Solanaceae* have been identified [8,15,29,30]. Here, we blasted nine Rops (called SlRop1–9) from the tomato genome database using OsRac1 as a query, which was the same as the ROPs reported by Dr. Zhao [29]. Phylogenetic relatedness uncovered that SlRops were categorized into 5 subclades. It is worth noting that SlRop3 falls into Group II together with OsRac1, OsRac4, and CaRop8, and these proteins are all regulators of immunity [15,29] (Figure 1B). Group-II HvRAC1 and HvROP6 played negative roles in barley resistance to powdery mildew fungi and HvRAC1 showed contrasting responses to rice blast fungus [30,44]. Additionally, TaRac1 in Group II from wheat modulates pathogen resistance through promoting lignin biosynthesis [45]. Similarly, SlRop4, 5, 6 and 7 were sorted into Group IV, where CaRop1, OsRac5, OsRac6, AtRop1, and AtRop6 were also present (Figure 1B). Intriguingly, the proteins mentioned above were also key factors in the plant immune system [15,32,33,40]. Furthermore, Group IV-belonging HvRAC3 and HvRACB negatively functioned in the defense against barley powdery mildew [30]. Therefore, it seems that the ROPs in Groups II and IV were likely to respond to defenses, but the ROPs in Groups I, III, and V were rarely involved in plant immunity. 

Our amino acid sequence analysis revealed that tomato ROPs with CaaL motif or GC-CG box in their PBRs were further divided into two types (Figure 2A). Although eight SlRops except SlRop8 terminate with a typical NLS sequence in their PBRs, not all of them can enter the nucleus. Membrane association is reportedly important for ROP function. The plasma membrane attachment of HvRAC3 and HvRACB was required for their accurate function during barley powdery mildew attack [30]. In rice, the activation of OsRacs promoted their plasma membrane localization, indicating that the plasma membrane was the place where Racs function for downstream events [15]. Subcellular localization of SlRop1–9 showed that they were all membrane-associated proteins, especially SlRop1 and SlRop3. The activation states of ROPs were not found to have any obvious effect on their membrane accumulation (Figure 2B). Therefore, it was possible that the natural intrinsic frequency of plasma membrane accumulation played a crucial role in the function of tomato Rac/Rop small GTPases.

Our results of HR induction by different activation levels of SlRop1–9 showed that only seven ROPs (SlRop3–9) from tomato can activate immune responses, including cell death and ROS burst (Figure 4 and Figure 5). First, we could not ignore the possibility that the inability of SlRop1 and 2 to trigger HR may be partially caused by the protein expression level because the amounts of SlRop1 and 2 were relatively low compared to the others (Figure S5B). Next, this result was also supported by the cases in barley Rho-like small GTPases in which only CA-HvRAC1 enhanced the whole H_2_O_2_ accumulation, but neither the active form of HvRACB or HvRAC3 induced it upon infection by *Bgh* [44]. It is well known that ROS production is mediated by NADPH oxidases in both animals and plants [42]. The rice small GTPase OsRac1 interacts with the N-terminal fragment of OsRBOHB to facilitate ROS accumulation [19,46]. DN-AtRop1 mediates resistance to *P. infestans* by inducing H_2_O_2_ accumulation through StRBOHD, a potato NADPH oxidase [32]. Therefore, it was highly possible that Rho-like small GTPases in tomato shared a similar way to produce ROS, and differences in the binding affinities or activation abilities of SlRops towards tomato OsRBOHB homologues ledto differences in ROS production. In addition, the phylogeny and conserved motif analysis revealed that both SlRop1 and 2 were present in an earlier branch of the phylogenetic tree and contained an extra motif 6 at their C-terminus (Figure S3). Speculatively, during the Rac/Rop small G protein evolutionary history, ROPs gained immune functions under natural selection pressures. Alternatively, ROPs were originally endowed with different characteristics to maintain diverse biological processes.

The disease resistance assay revealed that SlRop3 played a significant role in the defense against *R. solanacearum* and that SlRop4 made minor contributions (Figure 7). It was confusing that the active SlRop5–9 had the ability to boost HR but did not show any resistance to *R. solanacearum*. Similarly, CA-HvRAC1 triggered peroxide burst but also supported the penetration of biotrophic *Bgh* [44]. Downstream interactors of Rac/Rop small GTPases played critical roles in the defense system by regulating multiple responses against pathogens, such as the expression of pathogenesis-related genes and the biosynthesis of lignin [21,23,24,35]. Therefore, the variable downstream molecules targeted by SlRops might lead to different outputs when infected by *R. solanacearum*, which causes tomato plants to display varying degrees of insusceptibility.

In this study, we identified SlRop3 as a major positive regulator of defense against tomato bacterial wilt disease. Surprisingly, SlRop3 was previously reported to play an important role in resistance to *P. capsici* as well [29]. Since SlRop3 had already been found to be involved in resistance to both bacteria and oomycetes, it could serve as a target for gene editing and breeding to generate novel varieties with high resistance to tomato diseases in the near future. Collinear analysis showed that only SlRop3 had collinearity with all three species (Arabidopsis, pepper, and rice) (Figure S2B), indicating that SlRop3 might be much more conserved than other SlRops and retain important functions from ancestor.

## 4. Materials and Methods

### 4.1. Plant Materials and Growth

*Solanum lycopersicum* (cultivar *Ailsa Craig*) and *N. benthamiana* were used in this study. Tomato seeds were presoaked in water and shaken at 28 °C and 200 rpm for two days. Then these seeds were evenly spread on wet filter papers in glass petri dishes and germinated in an incubator with 28 °C, 75% relative humidity (RH), and 12-h photoperiod, for approximately one week. The seven-day-old tomato seedlings were ready for subsequent infection assays. For *N. benthamiana*, the seeds were directly sown in soil containing a mixture of peat and vermiculite (1:1.5, *v/v*) and allowed to germinate in a growth room with 25 °C, 65% RH, and 16-h photoperiod. Seven to ten days later, each seedling was transplanted to individual pots and continued to grow under the same conditions as those used for germination for four weeks. This step was followed by agroinfiltration.

### 4.2. RNA Isolation and qRT-PCR

Total mRNAs were extracted from different tomato samples using Total RNA Extraction Reagent (R401-01, Vazyme, Nanjing, China). 500 ng of RNA from each sample was used for reverse transcription reactions with a kit produced by Vazyme company (R223-01) according to the manual. The synthesized cDNA was subjected to quantitative analysis by amplification using ChamQ Universal SYBR qPCR Master Mix (Vazyme, Q711-02/03) on a CFX96 Touch Real-Time PCR machine (Bio-Rad, Hercules, CA, USA). *SlUbiquitin7* served as an internal control for data normalization. The primers sequences used for qRT-PCR were shown in Supplementary Table S1.

### 4.3. Plasmid Construction

The cDNAs of tomato were used as a template for amplifying the full-length CDS of the nine *SlRops*. The open reading frames (ORFs) of *SlRop1*–*9* were cloned into the pENTR/D-TOPO vector (Invitrogen, Carlsbad, CA, USA). The CA and DN forms of *SlRop1*–*9* were generated by site-directed mutagenesis using an overlapping PCR amplification method with primers possessing specific mutations and wild-type pENTR-SlRops as templates. The mutagenic sites were marked in Figure S1: Glycine (G) was changed to valine (V) to make CA mutants, and threonine (T) was mutated to asparagine (N) to produce DN mutants. The fragments of interest within the pENTR vector were then transferred into Gateway system destination vectors, including pGWB502-GW and pGWB542-YFP-GW, by LR reactions. The primers used for normal PCR and mutagenesis were also listed in Supplementary Table S1.

### 4.4. Transient Expression in N. benthamiana

Agroinfiltration of *N. benthamiana* was performed as described previously [25]. The binary plasmids inserted with target genes were first transformed into *Agrobacterium tumefaciens* strain GV3101 competent cells. Then, *A. tumefaciens* cells containing different recombinant plasmids were grown at 28 °C and 200 rpm overnight to obtain a density at 600 nm (OD_600_) of 0.8–1.0. The cells were pelleted by centrifugation, resuspended in infiltration buffer (10 mM MgCl_2_, 10 mM MES-NaOH (pH 5.6), and 150 µM acetosyringone), and adjusted to OD_600_ = 1.0. *A. tumefaciens* harboring the vector encoding the silencing suppressor P19 was always used to enhance exogenous gene expression in tobacco cells. In our cases, we mixed *Agrobacterium* carrying the appropriate constructs and P19 at a concentration ratio of 1:1, and incubated the mixture at 28 °C in the dark for at least 2 h before infiltration. Normally, the third and fourth leaves counting from the top of each plant were selected for injection, and agroinfiltrated plants were kept in the same growth room for two more days before sampling.

### 4.5. Subcellular Localization

YFP-SlRops WT and mutants were transiently overexpressed in *N. benthamiana* cells according to the protocol described above. Fluorescence images were captured under a Leica TCS-SP8 confocal microscope (Wetzlar, Germany) with a 64× oil-immersion objective. The laser line of the excitation wavelength was set to 514 nm, and the emission was collected over the range between 525 and 575 nm to image YFP.

### 4.6. Cell Fractionation and Quantification

Liquid nitrogen-frozen tobacco leaves (about 100 mg) were ground to fine powder with a pestle and mortar, and proteins were extracted and separated into membrane and soluble protein fractions as reported previously [47]. Anti-GFP (ab6556, Abcam, Cambridge, UK), anti-cAPX (AS06 180: cytosol; Agrisera, Vännäs, Sweden) and anti-H^+^ATPase (AS07 260: plasma membrane; Agrisera, Sweden) antibodies were used for subsequent immunoblotting analysis.

For cellular distribution quantification, the intensity of membrane (M_In_) and cytosolic (S_In_) fractions were obtained by measuring western blot bands from cell fraction assays with ImageJ software (National Institutes of Health, Bethesda, MD, USA). Due to the three times enrichment during fractionation, then M_In_ was divided by three to get M_In_’. Finally, the distribution ratios of M and S were calculated using the following formula:

M% = M_In_’/(M_In_’ + S_In_)

S% = S_In_/(M_In_’ + S_In_)

respectively.

### 4.7. HR Detection and Quantification

Agroinfiltrations of tag-free SlRops were performed as mentioned above. Each set of agrobacterial samples was separately infiltrated into the back of at least fifteen tobacco leaves with circles (approximately 1.5 cm diameter) for biological repeats. After five days, localized cell death symptoms were visible on the leaf surface and photographed with a high-resolution camera. Three independent experiments were conducted.

Cell death was further quantitatively assayed by measuring ion leakage derived from dead cells in tobacco leaves [48]. Similarly, by 5 dpi, the injected leaves were first detached from the tobacco plants, washed in water, and dried gently with tissue paper. Then five leaf discs with a diameter of 1 cm were sampled from five replicated leaves of each set and were floated on 5 mL of distilled water using a 50 mL-scale tube at room temperature. Three hours later, the floating leaf discs were carefully picked out and kept in a clean plastic dish. Immediately after, the electrical conductivity of the bathing solution was detected with a conductivity meter (METTLER TOLEDO, FE38-Standard) and recorded as “Value A”. The leaf discs were returned to the bathing solution and kept floating again. The tubes containing samples were sealed tightly, incubated at 100 °C water bath for 25 min, and allowed to cool down to room temperature naturally. Next the leaf discs were discarded, and the bath water was measured again to yield “Value B”. “Value A” and “Value B” were transferred to an Excel sheet and computed using a formula (= (Value A/Value B) × 100) to represent the percentage of electrolyte leakage. The experiments were repeated three times independently.

For the ROS detection assay, the infiltrated leaves overexpressing SlRops mutants or negative control Empty (pGWB502 empty vector) were immersed in 1 mg/mL DAB solution for 4 h at room temperature at 5 dpi. To observe ROS in situ, the leaves were then decolorized with 100% ethanol in a 55 °C incubator for 15 min. This step was repeated two to three times until the chlorophyll was completely removed. The depigmented leaves were washed with water and photographed. ROS production of each sample was quantified by measuring the pixel intensities of the infected regions using ImageJ software. The mean pixel intensity from three spots outside the infiltrated regions on each leaf was used to subtract the background.

### 4.8. R. solanacearum Strains, Growth, and Infection

The GMI1000 and Y45 strains of *R. solanacearum* were used to infect tomato and *N. benthamiana*, respectively [43,49]. Y45 and GMI1000 were cultured on Phi medium with/without tetracycline hydrochloride at 28 °C. The infection procedures for Y45 and GMI1000 were performed as described previously [43,50]. Briefly, fresh bacterial cultures were collected by centrifuging at 4000× *g* for 5 min, subsequently resuspended in sterile distilled water, and adjusted to concentrations at OD = 1 (approximately 10^9^ cfu/mL) for GMI1000 and 10^5^ cfu/mL for Y45. To infect the tomato seedlings, seven-day-old plants with similar growth rates were picked, and then the roots of selective seedlings were dipped in the GMI1000 inoculum for 1–2 s one by one. The inoculated seedlings were transferred to empty tubes and exposed to the air for 5 min. This step was followed by adding 1.5 mL sterile water to the empty tubes. The tested seedlings were kept in a growth chamber with high humidity (85–90%) for one week. The number of wilting plants was recorded every day to analyze the pathogenicity of GMI1000 in the tomato seedlings, and infected samples were harvested at the indicated time points to examine the posttranscriptional gene expression in response to *R. solanacearum*. To study the effects of SlRops on the virulence of *R. solanacearum*, we first infiltrated *A. tumefaciens* strain GV3101 pMP90 carrying the helper plasmid pSoup and single WT/CA/DN SlRop1–9 plasmids into 4-week-old *N. benthamiana* leaves. Appropriate concentrations of Y45 inoculum were injected into the same agroinfiltration region using a needleless syringe one day later. The infected plants were cultured for two days in a growth room at 75% RH and 27 °C. Four discs with a diameter of 4 mm from each sample were collected, weighed, and recorded. Then we added 100 µL of sterile distilled water to the tubes containing samples and homogenized the leaf discs by high-speed shaking with metal beads. The total leaf lysate was diluted 1000 times by using sterile distilled water and mixed thoroughly. A 50 µL mixture was spread on Phi medium with the appropriate antibiotics and incubated at 28 °C for 2 days. The number of colonies (cfu) on each plate was counted and further calculated in Excel 2020 using the formula: = PRODUCT (cfu/0.05, 100, 1/fresh weight (g)). Lastly, this value was converted by LOG10.

### 4.9. Western Blotting

Ten leaf discs with a 4-mm diameter were collected from each agroinfiltrated leaf, transferred to a 1.5-mL sterile tube with three metal beads, and frozen in liquid nitrogen rapidly. Then the samples were ground into a fine powder twice by a tissue grinder with a high shaking frequency of thirty oscillations/s for 1 min under extremely low temperature conditions. The total proteins were extracted by adding 250 µL of lysis buffer (50 mM Tris-HCl [pH 7.5], 150 mM NaCl, 10% glycerol, 0.5% NP-40, 5 mM EGTA, 5 mM DTT, and one tablet of EDTA-free protease inhibitor [Roche, Basel, Switzerland]). This step was followed by mixing vigorously and standing on ice for 30 min. The homogenate was centrifuged at 15,000× *g* for 15 min, and the resulting supernatant was transferred to a new centrifuge tube. The protein lysate was mixed with 4× SDS loading buffer, boiled for 10 min in a water bath, and subjected to standard SDS-PAGE analysis. For immunoblotting, anti-GFP antibody (Abcam, ab6556) was diluted at 1:10,000 before use. After exposure, the PVDF membrane was stained with Ponceau dye to visualize the bands of an internal control.

### 4.10. Sequence Alignment and Phylogenetic Analysis

The amino acid sequences of nine ROPs in tomato were aligned and visualized by using MUSCLE [51] and GeneDoc (http://www.nrbsc.org/gfx/genedoc/index.html, accessed on 12 October 2021), respectively. The conserved protein domain was predicted with the SMART server (https://smart.embl.de, accessed on 15 August 2021). The alignment sequences were trimmed using TrimAI [52] and then applied to build phylogenetic trees in IQ-TREE software [53] by setting one thousand bootstrap replicates.

### 4.11. Analysis of Chromosomal Mapping, Conserved Motif, Gene Structure, and Collinearity

The conserved motifs within the protein sequences were identified by using MEME software [54]. The visualizations of the chromosomal distribution and data combined with an evolutionary tree, conserved motifs, and diagrams of gene structure, were both achieved by using TBtools [55]. The collinearity analysis for SlRops among different plant species was detected with MCScanX [56].

### 4.12. Statistical Analysis

Means and SDs of all the graphs were automatically calculated using GraphPad Prism (version 9.2.0, San Diego, CA, USA). The statistical significance was analyzed with the one-way analysis of variance (ANOVA) program in GraphPad Prism.

### 4.13. Accession Numbers

*OsRac1*–*7*: Os01g0229400, Os05g0513800, Os02g0742200, Os06g0234200, Os02g0834000, Os02g0120800, and Os02g0312600;

*CaRop1*–*10*: DQ257288, CA00g82910, CA00g84620, CA01g27430, CA02g04310, CA02g05500, CA02g21300, CA03g28070, CA04g05500, and CA08g19280;

*AtAPSR1 and AtRop1*–*11*: At3g51290, At3g51300, At1g20090, At2g17800, At1g75840, At4g35950, At4g35020, At5g45970, At2g44690, At4g28950, At3g48040, and At5g62880.

## Supplementary Materials

The following supporting information can be downloaded at: https://www.mdpi.com/article/10.3390/ijms23179727/s1.
