# Supplementary material for: Comprehensive Analysis of Subcellular Localization, Immune Function and Role in Bacterial wilt Disease Resistance of Solanum lycopersicum Linn. ROP Family Small GTPases
Source: Int J Mol Sci. 2022 Aug 27;23(17):9727. doi: 10.3390/ijms23179727 (PMC9456112; doi:10.3390/ijms23179727)
Supplement: Supplementary file 1 [file ijms-23-09727-s001.zip › ijms-1825340-supplementary/Supplementary Table S1.pdf]

Table S1

Primers for pENTR cloning	
**Primer name**	**Sequence (5'-3')**
SlRop1-F	CACC ATGAATGCTTCAAAGTTCATC
SlRop1-R	CTAAGCAACACAGCCTCCACAAACG
SlRop2-F	CACC ATGAATGTTTCAAAGTTCATC
SlRop2-R	CTAAACAGCAGCACAGCCTCCACAT
SlRop3-F	CACC ATGGCCTCAAGTGCTTCAAGA
SlRop3-R	TCACTTTAAACAAACGAGCTTCCTT
SlRop4-F	CACC ATGAGTGCTTCTAGGTTTATA
SlRop4-R	TCACAAGATAGAGCAGGCTTTCTGA
SlRop5-F	CACC ATGAGTGCTTCAAGGTTTATC
SlRop5-R	TCACAAAATAGAACATGCCTTTTGG
SlRop6-F	CACC ATGAGCGCATCCAGGTTCATC
SlRop6-R	TCACAAAATTGAGCAGGCCCTTTGG
SlRop7-F	CACC ATGAGTGCTTCCAGGTTTATC
SlRop7-R	TTATAGTTGATCACAATATCGAGCA
SlRop8-F	CACC ATGACTACTGCTAGATTCATC
SlRop8-R	TCATAGAATTGCACATAACGTCCTC
SlRop9-F	CACC ATGAATAGTAGTACTAGTGCT
SlRop9-R	TCAAAGTATCCAGCAGGCTTTGTGT
**Primers for RT-PCR**	
**Primer name**	**Sequence (5'-3')**
SlRop1-qPCR-F	CTAGCAGATCATATGGGCTCAA
SlRop1-qPCR-R	GTTGAGTTTTAGAGCTGCACTC
SlRop2-qPCR-F	ACAACGCATATCTAGCTGATCA
SlRop2-qPCR-R	CTGCATTCTACATAAGCTGCAG
SlRop3-qPCR-F	TTCCCATCAATTCGGTCTCTAG
SlRop3-qPCR-R	TGTAGCAGAATTAGTCGCATCT
SlRop4-qPCR-F	GTTGTCCGTTTGATTATAGGCC
SlRop4-qPCR-R	ACACAACAACATTTAGCAGACG
SlRop5-qPCR-F	GTAAAGCCAGCTATGAAAACGT
SlRop5-qPCR-R	TTGTTCCAACAAGCACTATTGG
SlRop6-qPCR-F	GTTTCTGTTTTCCTTTTTGCGG
SlRop6-qPCR-R	CGAGGATAAAATAGAGTGGCCT
SlRop7-qPCR-F	CTTCTTCTTCGTTGGTTGACAG
SlRop7-qPCR-R	CGATGACCCACTTCTTATCAGA
SlRop8-qPCR-F	TAACTTCAGTGCAAATGTGGTG
SlRop8-qPCR-R	GACATCAGCTCCTCTGTAACTT

Table S1

Continued

**Primers for RT-PCR**		
**Primer name**	**Sequence (5'-3')**	
SlRop9-qPCR-F	ACTTTGATCTAAGTCGCTGGAA	
SlRop9-qPCR-R	AGCCAGCAATAAACTCAACAAG	
SlUBQ7-qPCR-F	GATCGTATCAAGGAACGTGTTG	
SlUBQ7-qPCR-R	CTTTAGCTGTCTTGTCATCTGC	
**Primers for Primers for mutagenesis**		
**Primer name**	**Sequence (5'-3')**	
SlRop1-G15V-F	TGTAGGCGATGTTGCTGTTGGCAAGACCTGCATGCTCA	CA
SlRop1-G15V-R	TGCCAACAGCAACATCGCCTACAGTTACACATTTGATG	
SlRop2-G15V-F	TGTAGGTGATGTAGCTGTTGGAAAGACATGTATGTTAA	
SlRop2-G15V-R	TTCCAACAGCTACATCACCTACAGTCACACATTTGATG	
SlRop3-G17V-F	GGTTGGTGATGTGGCTGTTGGCAAGACTTGTATGCTTA	
SlRop3-G17V-R	TGCCAACAGCCACATCACCAACCGTGACACATTTGATG	
SlRop4-G15V-F	CGTGGGCGATGTAGCTGTGGGTAAAACTTGTCTTCTCA	
SlRop4-G15V-R	TACCCACAGCTACATCGCCCACGGTAACACACTTTATA	
SlRop5-G15V-F	CGTCGGCGATGTTGCCGTTGGAAAGACGTGTCTCTTGA	
SlRop5-G15V-R	TTCCAACGGCAACATCGCCGACGGTAACACATTTGATA	
SlRop6-G15V-F	CGTTGGTGATGTTGCTGTTGGAAAGACATGCCTCTTGA	
SlRop6-G15V-R	TTCCAACAGCAACATCACCAACGGTGACACACTTGATG	
SlRop7-G15V-F	AGTGGGTGATGTTGCTGTTGGAAAGACTTGTCTTTTGA	
SlRop7-G15V-R	TTCCAACAGCAACATCACCCACTGTAACACACTTGATA	
SlRop8-G15V-F	TGTTGGTGATGTAGCTGTTGGGAAAACTTGTATGCTAA	
SlRop8-G15V-R	TCCCAACAGCTACATCACCAACAGTAACACATTTGATG	
SlRop9-G27V-F	TGTTGGAGATGTTGCTGTTGGAAAAACTTGTCTTCTCA	
SlRop9-G27V-R	TTCCAACAGCAACATCTCCAACAGTAACACATTTGATG	
SlRop1-T20N-F	TGTTGGCAAGAACTGCATGCTCATTTGTTACACCAGTA	DN
SlRop1-T20N-R	TGAGCATGCAGTTCTTGCCAACAGCACCATCGCCTACA	
SlRop2-T20N-F	TGTTGGAAAGAACTGTATGTTAATTTGTTACACTAATA	
SlRop2-T20N-R	TTAACATACAGTTCTTTCCAACAGCTCCATCACCTACA	
SlRop3-T22N-F	TGTTGGCAAGAATTGTATGCTTATTTGCTATACCAGTA	
SlRop3-T22N-R	TAAGCATACAATTCTTGCCAACAGCCCCATCACCAACC	
SlRop4-T20N-F	TGTGGGTAAAAATTGTCTTCTCATTTCGTATACCAGCA	
SlRop4-T20N-R	TGAGAAGACAATTTTTACCCACAGCTCCATCGCCCACG	
SlRop5-T20N-F	CGTTGGAAAGAACTGTCTCTTGATTTCTTACACCAGCA	
SlRop5-T20N-R	TCAAGAGACAGTTCTTTCCAACGGCACCATCGCCGACG	
SlRop6-T20N-F	TGTTGGAAAGAACTGCCTCTTGATTTCCTACACCAGCA	
SlRop6-T20N-R	TCAAGAGGCAGTTCTTTCCAACAGCACCATCACCAACG	

Table S1

Continued

Primers for Primers for mutagenesis		
**Primer name**	**Sequence (5’-3’)**	
SlRop7-T20N-F	TGTTGGAAAGAATTGTCTTTTGATTTCTTACACCAGCA	**DN**
SlRop7-T20N-R	TCAAAAGACAATTCTTTCCAACAGCACCATCACCCACT	
SlRop8-T20N-F	TGTTGGGAAAAATTGTATGCTAATTTCATACACTAGTA	
SlRop8-T20N-R	TTAGCATACAATTTTTCCCAACAGCTCCATCACCAACA	
SlRop9-T32N-F	TGTTGGAAAAAATTGTCTTCTCATCTCCTACACTAGCA	
SlRop9-T32N-R	TGAGAAGACAATTTTTTCCAACAGCACCATCTCCAACA	
SlRop3-2B-F	GAAAAAACAAGAAGAGGGATGTCTCATGAATGTGATGTGCG	**-2B**
SlRop3-2B-R	TGAGACATCCCTCTTCTTGTTTTTTCTTCTCCTTTTGCTTT	
SlRop3-3B-F	GCAAAAGGAGGAGGAAGAACAACGTCGGGGATGTCTCATGAATG	**-3B**
SlRop3-3B-R	CCCGACGTTGTTCTTCCTCCTCCTTTTGCTTTTGTGGTGGCTTG	
SlRop3+L-F	TTGTTTAAAGCTATGAAAGGGTGGGCGCGCCGACCCAG	**+L**
SlRop3+L-R	CACCCTTTCATAGCTTTAAACAAACGAGCTTCCTTCCG	
