# Supplementary material for: Comprehensive Analysis of Subcellular Localization, Immune Function and Role in Bacterial wilt Disease Resistance of Solanum lycopersicum Linn. ROP Family Small GTPases
Source: Int J Mol Sci. 2022 Aug 27;23(17):9727. doi: 10.3390/ijms23179727 (PMC9456112; doi:10.3390/ijms23179727)
Supplement: Supplementary file 1 [file ijms-23-09727-s001.zip › ijms-1825340-supplementary/Supplementary text.pdf]

Supplementary information includes the following items:

1. Six supplementary figures and legends.

Figures S1, S2, and S3 are related to Figure 1.

Figures S4 and S5 are related to Figure 2.

Figure S6 is related to Figure 6.

2. One supplementary table

Table S1. List of primers for experimental procedures

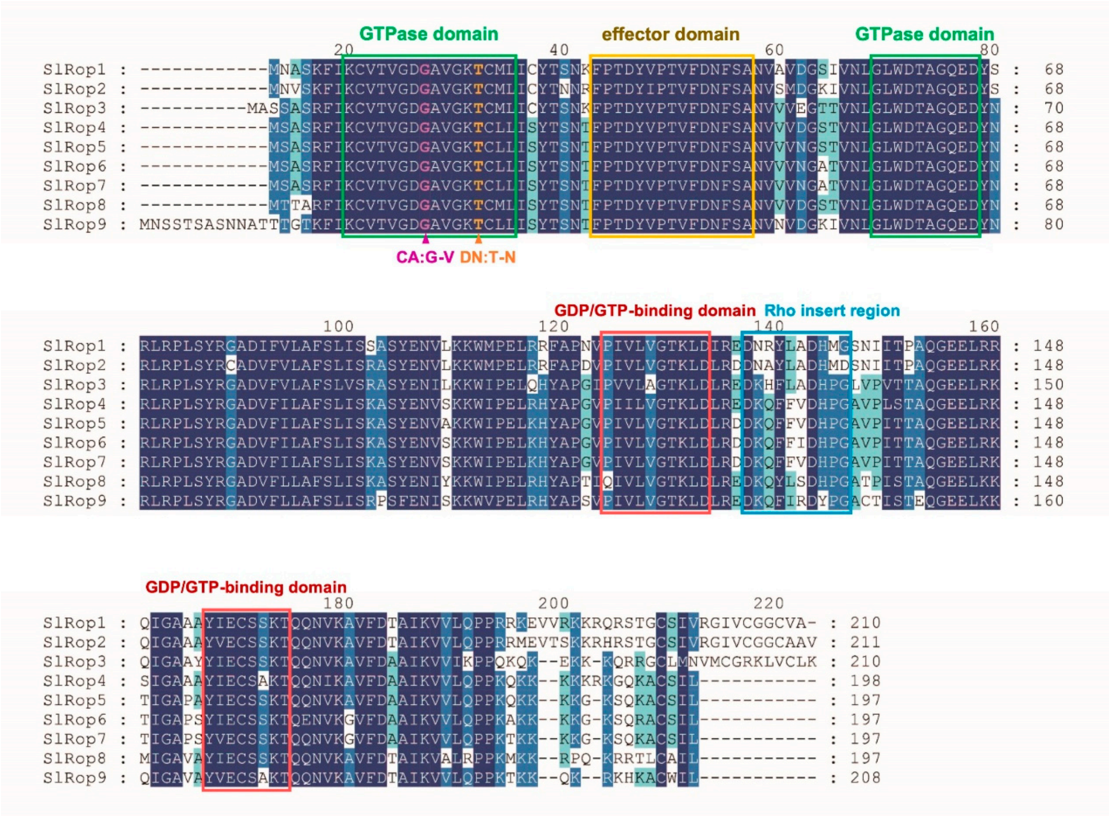

Figure S1. Multiple sequence alignment of Rac/Rop family proteins in tomato.
The backgrounds with different shades of blue reflect the relative degrees of conservation of each amino acid residue. Colored boxes represent conserved domains with varied functions in SlRops. The residues used to generate the CA and DN mutants of SlRops are highlighted in magenta and orange, respectively. CA: G-V, indicating glycine (G) was substituted with valine (V) to make CA mutants; DN: T-N, indicating threonine (T) was changed to asparagine (N) to generate DN mutants.

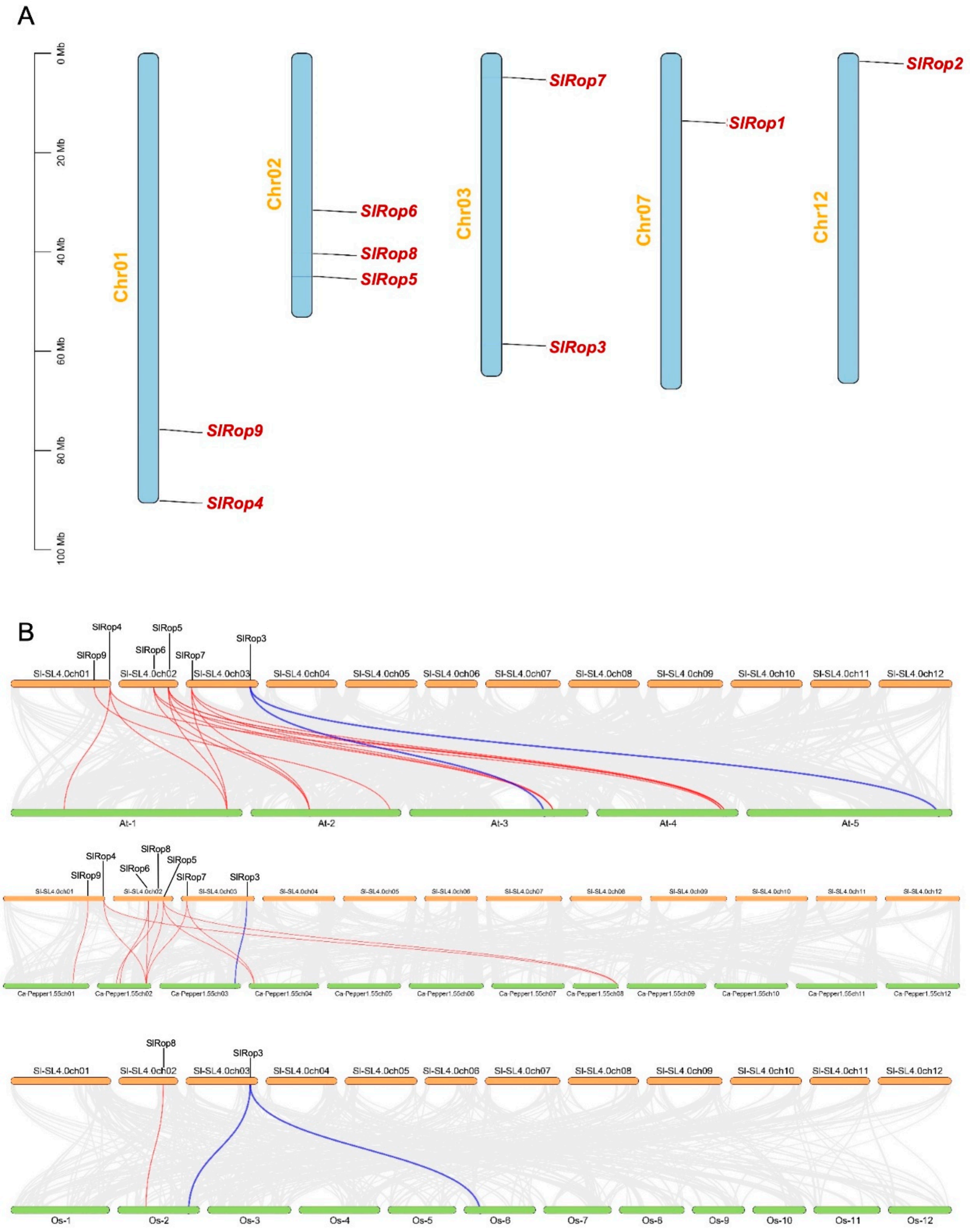

Figure S2. Chromosomal location and collinearity of tomato *Rac/Rop* family genes.
A, Chromosomal distribution of *SlRops* genes in tomato genomes. The chromosome numbers are displayed on the left side of the blue sticks, while the approximate location of each *SlRop* gene on the corresponding chromosome is marked on the right side. The scale bar on the left represents the length of chromosome in megabases (Mb). **B**, Interspecies collinearity analysis of SlRop1–9 among Arabidopsis, pepper, and rice. The chromosome numbers and gene names are shown outside. Abbreviations: Sl, Solanum lycopersicum (tomato); At, Arabidopsis thaliana (Arabidopsis); Ca, Capsicum annuum (pepper); Os, Oryza sativa (rice). Blue and red lines represent the syntenic relationships of SlRop3 and the remaining SlRops with other plant species, respectively.

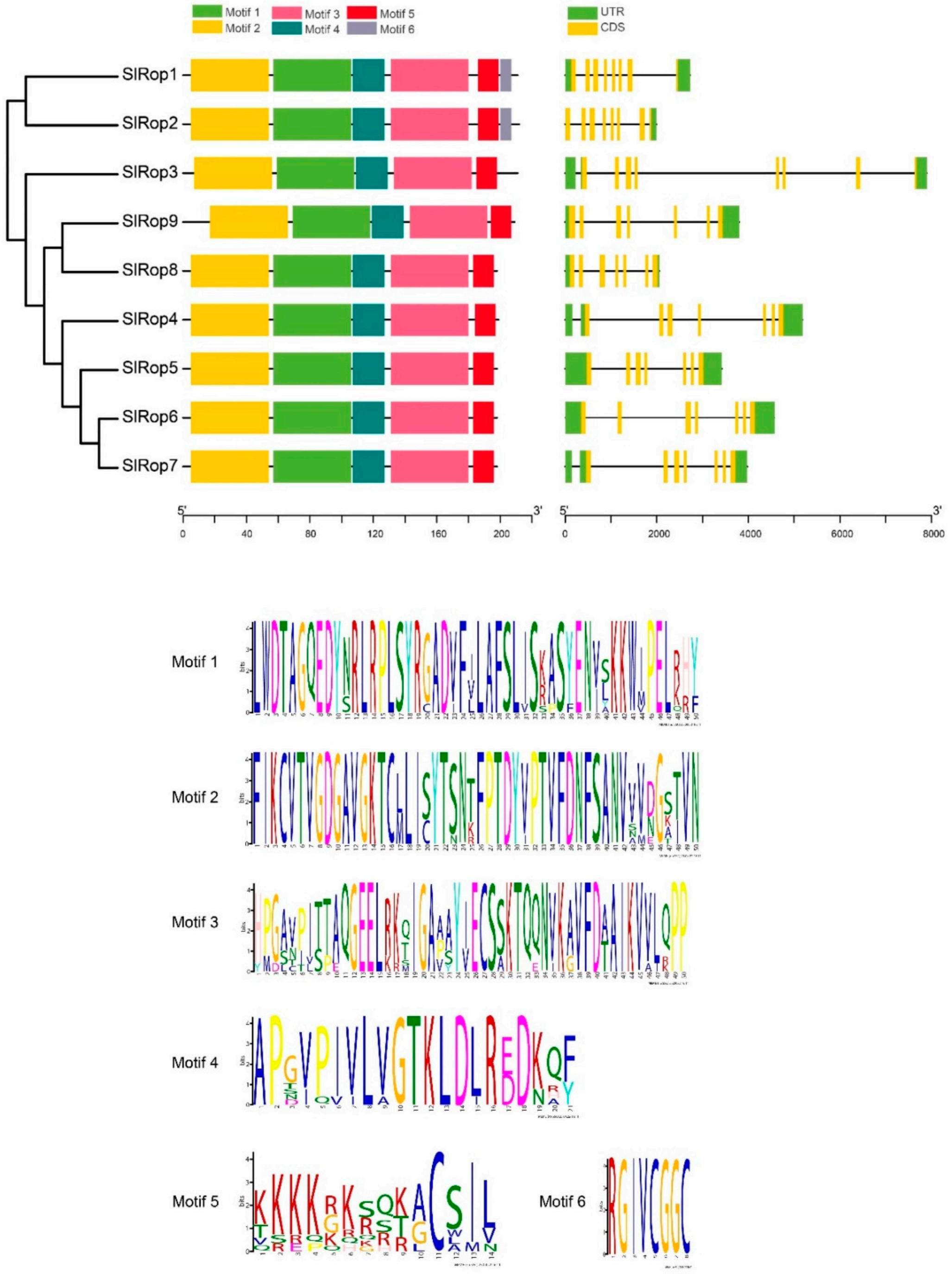

Figure S3. Conserved motif and gene structure analysis of SlRop1–9

Phylogenetic tree, conserved motif, and gene structure of Rac/Rop family small GTPases in tomato. The conserved motifs are denoted by boxes with different colors in the middle panel. The left panel indicates the gene structures of nine *SlRops*, in which green rectangles, yellow rectangles, and black lines represent the regions of UTRs, CDSs, and introns, respectively. The scale bars at the bottom of the sketch maps show the lengths of amino acids and nucleic acids, respectively. Sequence logos display the amino acid composition of six conserved motifs in tomato ROPs.

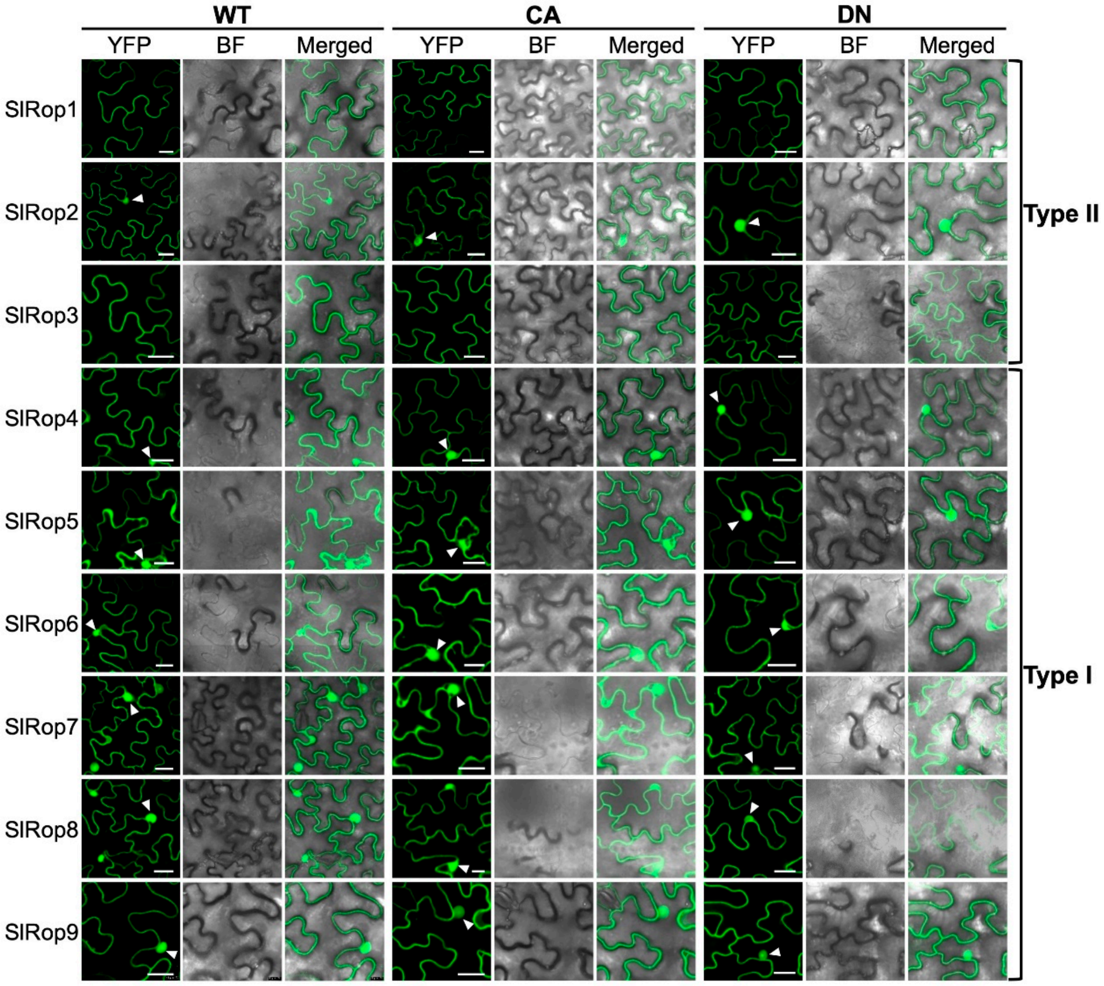

Figure S4. Localization of YFP-SlRops in *N. benthamiana*

Subcellular distribution of tomato Rho-like small GTPases in *N. benthamiana*. Agrobacterium carrying YFP-fused SlRop1–9 wild type and mutants were individually injected into tobacco leaves. Fluorescent signals were detected in the epidermal cells on the abaxial side of the infiltrated leaves at 2 dpi. White arrows represent the nucleus. WT, wild type; CA, constitutively active; DN, dominant-negative; BF, bright field. Scale bars, 25 μm. The experiment was repeated twice.

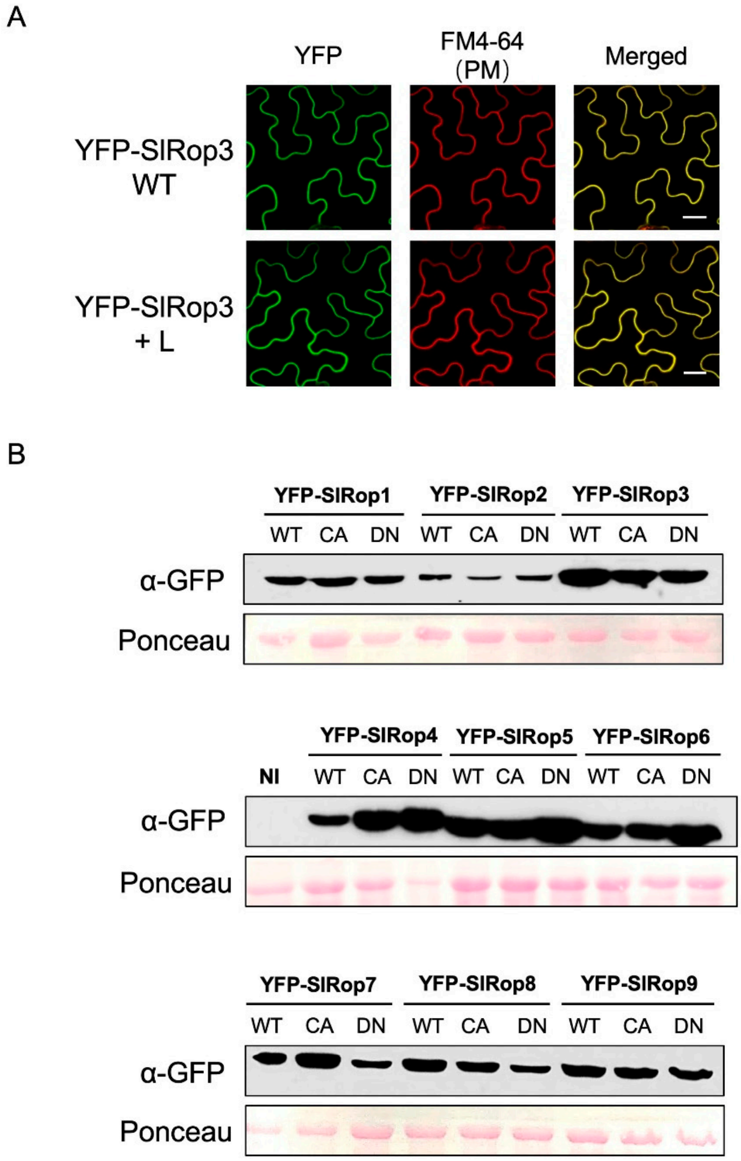

Figure S5. Localization of CaaL motif-containing SlRop3 mutant and protein accumulation levels of SlRops

A, Tobacco leaves were injected with Agrobacterium carrying YFP-SlRop3 mutants (green) and stained with FM4-64 (red: plasma membrane marker). Scale bars, 25 µm. The experiment was repeated twice. **B**, YFP-tagged SlRop1–9 WT and mutants were transiently expressed in *N. benthamiana*. By 2 dpi, the total proteins in agroinfiltrated leaves were extracted and subjected to immunoblotting analysis with anti-GFP antibody. NI, non-infiltrated sample, was used as a negative control. The post-transfer PVDF membrane was stained with Ponceau S and served as an internal control. The results of three membranes were obtained at the same time and repeated independently at least two times.

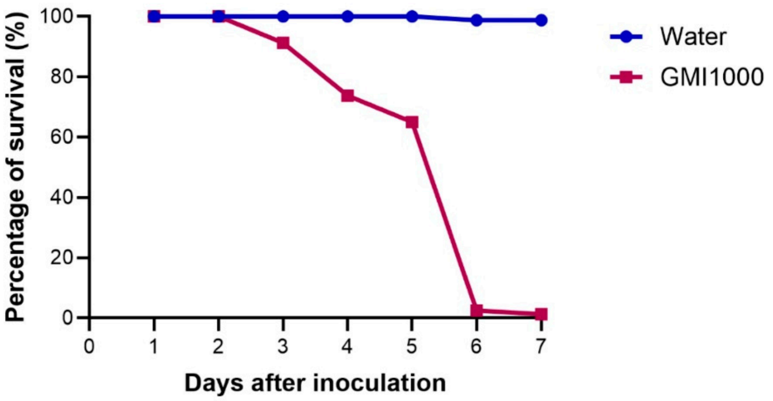

Figure S6. Virulence of GMI1000 on tomato cultivar *Ailsa Craig*

Virulence data of the GMI1000 strain on root-inoculated tomato seedlings. The x-axis demonstrates the days after inoculation, and the y-axis represents the survival percentage of tomato seedlings. The experiment was repeated at least three times.
